# Immigrant background and medicine use for aches: national representative study of adolescents

**DOI:** 10.1186/2052-3211-7-1

**Published:** 2014-01-22

**Authors:** Lourdes Cantarero-Arévalo, Bjørn E Holstein, Anette Andersen, Maria Kristiansen, Ebba H Hansen

**Affiliations:** 1 Department of Pharmacy, Section for Social and Clinical Pharmacy. Faculty of Health and Medical Sciences, University of Copenhagen, Universitetsparken 2.2100, Copenhagen, Denmark; 2 National Institute of Public Health, University of Southern Denmark, Copenhagen, Denmark; 3 Danish Research Centre for Migration, Ethnicity and Health. Faculty of Health and Medical Sciences, University of Copenhagen, Copenhagen, Denmark

**Keywords:** Adolescents, Pharmacoepidemiology, Immigrants, Denmark, School health, Ethnicity

## Abstract

**Objectives:**

The aims of the study were to examine the association between immigrant background and medicine use for headache and stomach-ache among adolescents, and whether symptoms of headache and stomach-ache could explain the differences in medicine use.

**Methods:**

We used data from the Danish contribution to the WHO-affiliated international cross-sectional survey Health Behaviour in School-aged Children (HBSC) in 2006. Among boys, a total of 4170 ethnic Danes, 244 descendants of immigrants, and 224 immigrants participated. Among girls, 4310 ethnic Danes, 264 descendants of immigrants, and 232 immigrants were included. The associations between migrant background and medicine use for headache and stomach-ache by means of multilevel multivariate logistic regression analyses adjusted for age group, symptoms and the clustering effect of school and stratified by sex due to interactions.

**Results:**

Among boys, the risk of medicine use for stomach-ache was higher for immigrants (odds ratio (OR), 1.54; 95% confidence intervals (CI), 0.99-2.44)) and descendants (OR, 1.97 (1.33-2.94)) compared to ethnic Danes. Similar associations were found for use of medicine for stomach-ache for immigrant girls (OR, 1.55 (1.12-2.15) and use of medicine for headache among boys (immigrants (OR, 1.36 (1.02-1.97 and descendants (1.48 (1.12-1.97)). Symptoms of aches were all independently associated with medicine use. After adjusting for these factors the association between immigrant background and medicine use attenuated slightly.

**Conclusion:**

Among adolescents in Denmark, the risk of medicine use for headache and stomach-ache was higher for immigrants and descendants as compared to ethnic Danes, with the exception of medicine use for headache among girls.

## Introduction

In the Western world, high proportions of adolescents use medicines for aches [1,2]. The proportion of young people who use medicine for aches varies substantially across countries and this variation cannot be explained by frequency of symptoms alone [1,2]. Adolescents’ medicine use for aches is a public health concern because it is likely that many adolescents use medicines outside formal therapeutic indication, e.g. as a way to deal with daily stressors [3].

A review of 6- to 17-year-old healthy children’s perceptions of medicines showed a high autonomy in using medicines which was found to be disturbing given their poor knowledge of medicine [4]. Medicine use for aches among adolescents is sensitive to a range of socio-demographic and psychosocial circumstances, e.g. age, sex, and adverse living conditions [5]. And a longitudinal study suggests that medicine use in adolescence predicts medicine use in young adulthood [6]. The most important predictor of adolescents medicine use for aches seems to be the prevalence of aches [1,2]. Further, this kind of medicine use is associated with indicators of stressors such as low social class [5] and exposure to bullying [7].

In terms of migration, Denmark is a fairly homogeneous country with only 7% of children born in Denmark with foreign ancestry and 3% immigrant children. The proportion of children with immigrant background is rising. There is little information about medicine use for aches in relation to immigrant background. We have only been able to identify two studies about immigrant background and medicine use for aches among adolescents, one short communication which suggests that adolescents from immigrant families use medicines more often than the majority population [8], and a study which suggests that self-medication was not related to migrant background [9]. Adolescents with immigrant background are more likely be exposed to a number of psychosocial stressors (e.g. related to belonging to a minority group and being raised in multicultural settings challenging their notions of identity) which may cause them to be at higher risk of using medicine beyond what may be explained by aches [10].

In this study, we use data from a national representative survey among populations of 11-, 13- and 15-year-old adolescents living in Denmark in 2006 in order to examine whether there is an association between immigrant background and medicine use for aches among adolescents. We furthermore investigate whether differences in prevalence of aches explain differences in medicine use between children with immigrant background and the majority population.

## Methods

### Setting

Denmark has traditionally been a culturally homogeneous country. Immigration from non-Western countries was almost non-existent before the 1960s where the first wave of immigrants from Turkey filled a gap in the labour force. Later waves of immigrants from Lebanon, Iraq, Afghanistan and Somalia came to Denmark for political reasons in the 1990s and the beginning of this century. In 2012, 23,099 Non-Western immigrants and 92,880 descendant children with Non-Western ancestry were living in Denmark, or 1.9% and 7.7% respectively of a total population of approximately 1.2 million children (extracted from Statistics Denmark, Statistics Bank, October 2012). Although there are no official estimates, the number of non-documented immigrant children is generally considered to be very small.

Among immigrants, the largest populations of children are from Iraq, Afghanistan and Somalia. Among descendants, the three largest groups are constituted by children with Turkish, Lebanese and Iraq ancestry.

### Study population

We used data from the Danish contribution to the WHO-affiliated international cross-sectional survey Health Behaviour in School-aged Children (HBSC) in 2006 [11]. The study population includes pupils who attended ordinary public schools (not schools for pupils with special needs) in three age groups, (11-, 13-, and 15-years), corresponding to the 5^th^, 7^th^ and 9^th^ grade in the Danish school system. The sampling was stratified to ensure a sufficient share of pupils from rural regions. A total of 127 schools received an invitation to participate in the study and 107 participated (84%) while the remaining schools declined participation, mostly due to involvement in other health surveys. The response rate was 99.0% of pupils present on the day of data collection, corresponding to 89.2% of the pupils formally enrolled in the participating classes, yielding a total of 9514 pupils. Among boys, a total of 4170 ethnic Danes, 244 descendants of immigrants, and 224 immigrants participated. Among girls, 4310 ethnic Danes, 264 descendants of immigrants, and 232 immigrants were included.

### Data collection

The participating pupils completed the internationally standardized HBSC [12] questionnaire in the classroom after instruction from a teacher. They returned the completed questionnaire to their teacher in sealed envelopes to protect anonymity, and no school staff members had access to the questionnaires.

### Measurements

The outcome measure was the HBSC questions on medicine use behaviour: "In the last month: did you take tablets or medicines for the following? 1) headache, and 2) stomach-ache". The response code was "yes, several times", "yes, once", "no". Very few pupils used the first response category and we dichotomized the responses into "yes" and "no". We did not ask about specific medications as our study concerns medicine use behaviour.

The independent variable "immigrant background" was measured by the following items: 1) "Were you born in Denmark?", 2) "In which country was your mother born?", 3) "In which country was your father born?" Based on these data, the students were categorised into 1) ethnic Dane, 2) immigrant, 3) descendant of immigrants, and 4) unknown. We followed the definition of immigrant background as used by Statistics Denmark. An *immigrant* is a person born abroad, both of whose parents (or one of them if there is no available information on the other parent) are foreign citizens or born abroad. If there is no available information on either of the parents and the person was born abroad, the person is also defined as an immigrant. *A descendant of immigrants* (hereinafter called descendant) is defined as a person born in Denmark whose parents (or one of them if there is no information on the other parent) are either immigrants or descendants. An *ethnic Dane* is a person born in Denmark whose parents are born in Denmark.

As symptoms are the strongest predictor for adolescents’ medicine use [7], we included prevalence of headache and stomach-ache in the analyses. Symptom prevalence was measured by two items from the validated HBSC Symptoms Check List as follows: "During the last 6 months, how often have you experienced 1) headache and 2) stomach-ache?" (13). The responses were recoded into two levels namely, 1) at least monthly and 2) seldom and never.

### Statistical procedures

Participants with missing data on migration status were not included in the analyses (36 boys and 34 girls). The percentage of missing data for medicine use was in the range of 3.1-6.8% for girls, and 2.7-5.4% for boys. The percentage of missing data for symptoms was in the range of 1.2%-1.8% for girls and 1.1-2.4% for boys. The analysis was sex-stratified due to interaction between age and sex. The final study population was 4674 boys and 4840 girls.

Differences in prevalence of medicine use between ethnic Danes, immigrants and descendants were analyzed using Chi-square test. We estimated the associations between immigrant background and medicine use for headache and stomach-ache by means of multivariate logistic regression analyses adjusted for age group. The analyses were stratified by sex due to interaction between sex and immigrant background and by frequency of symptoms to examine the variation between different categories. Type 3 analyses were included to better understand the statistically significant power of the variables included in the analysis, considering different levels of dose–response. Finally, we adjusted all model for the clustering of pupils due to the sampling of schools and not individuals. Multilevel logistic regressions were implemented using the SAS procedure *proc glimmix*. All statistical analysis was performed using SAS version 9.2.

### Ethical considerations

In Denmark, ethical approval of population-based surveys is not required and there is no agency to deal with this issue. Therefore, the researchers asked for approval and consent from the school management, the board of parents, and the board of pupils in every participating school. The students got oral and written information about the study and that participation was voluntary and completely anonymous.

## Results

Table 1 shows the prevalence of medicine use and symptoms according to immigrant background (immigrants or descendants) compared to ethnic Danes. For both boys and girls, the percentage of the study population with immigrant background was approximately 10%. With the exception of medicine use for headache among girls, the prevalence of medicine use was higher for immigrants and descendants than ethnic Danes.

**Table 1:** Characteristic of the study population: prevalence of outcome and explanatory factors

**Boys (n = 4674)**					
	Ethnic Danes	Descendants	*Chi-sq test p value for similarity with ethnic Danes*	Immigrants	*Chi-sq test p value for similarity with ethnic Danes*
	(n = 4170)	(n = 244)		(n = 224)	
**Last month prevalence of medicine use for (%)**					
Headache	37.6	43.5	*0.0734*	44.4	*0.0456*
Stomach-ache	8.3	16.4	*<.0001*	12.1	*0.0615*
**Reported symptoms monthly or more (%)**					
Headache	38.1	32.2	*0.0654*	38.0	*0.9633*
Stomach-ache	25.9	31.9	*0.0426*	29.3	*0.2724*
**Girls (n = 4840)**					
	Danes	Descendants	*Chi-sq test p value for similarity with ethnic Danes*	Immigrants	*Chi-sq test p value for similarity with ethnic Danes*
	(n = 4310)	(n = 264)		(n = 232)	
**Last month prevalence of medicine use for (%)**					
Headache	51.1	47.4	*0.2456*	54.7	*0.3009*
Stomach-ache	21.6	27.0	*0.0487*	30.4	*0.0025*
**Reported symptoms monthly or more (%)**					
Headache	51.0	46.4	*0.1555*	47.3	*0.2772*
Stomach-ache	46.9	50.9	*0.2115*	51.5	*0.1778*

### Symptoms of aches

There were no major differences in prevalence of headache and stomach-ache (Table 1). Among boys, the age group adjusted odds ratio (OR) of symptoms of headache was 0.76 (95% CI: 0.58-1.01) for descendants and 0.99 (95% CI: 0.74-1.30) for immigrants compared to ethnic Danes and the age group adjusted OR of symptoms of stomach-ache was 1.31 (95% CI: 0.99-1.75) for descendants and 1.20 (95% CI: 0.88-1.62) for immigrants compared to ethnic Danes. Among girls, the age group adjusted OR of symptoms of headache was 0.84 (95% CI: 0.65-1.08) for descendants and 0.83 (95% CI: 0.64-1.10) for immigrants compared to ethnic Danes and the age group adjusted OR of symptoms of stomach-ache was 1.20 (95% CI: 0.93-1.56) for descendants and 1.16 (95% CI: 0.88-1.53) for immigrants compared to ethnic Danes (results not shown).

### Medicine use for headache

Table 2 shows the association between medicine use for headache and immigrant background. Among boys, there was a higher risk of medicine use among immigrants and descendants which did not attenuate after adjusting for symptoms and the clustering effect of school in the analysis. Symptoms of headache were significantly associated with medicine use. Among girls there were no differences in use of medicine for headache according to immigrant background whereas symptoms were significantly associated with use of medicine.

**Table 2:** Odds Ratio (OR) with 95% confidence interval (CI) for self-reported medicine use for headache by migrant background, stratified by sex

	**Medicine use for headache**	
	**Model 1**	**Model 2**
**Boys**		
Ethnic Dane	1	1
Descendant	1.31 (1.00-1.73)	1.48 (1.10-1.97)
Immigrant	1.30 (0.98-1.71)	1.36 (1.02-1.87)
*Type 3 analysis*	*0.0327*	*0.0074*
**Headache**		5.29 (4.59-5.98)
**Girls**		
Ethnic Dane	1	1
Descendant	0.83 (0.63-1.10)	0.88 (0.68-1.20)
Immigrants	1.08 (0.81-1.43)	1.19 (0.91-1.66)
*Type 3 analysis*	*0.3689*	*0.3538*
**Headache**		4.92 (4.36-5.61)

Model 1: adjusted for age group (Intra Class Correlation (ICC)) = 0.0093 for boys; 0.0223 for girls).

Model 2: adjusted for age group, and headache (ICC = 0.0143 for boys; 0.0190 for girls).

### Medicine use for stomach-ache

Among boys and girls there was a relationship between medicine use for stomach-ache and immigrant background which persisted after adjusting for symptoms, even though the association was attenuated slightly (Table 3).

**Table 3:** Odds Ratio (OR) with 95% confidence interval (CI) for self-reported medicine use for stomach-ache by ethnic background, stratified by sex

	**Medicine use for stomach-ache**	
	**Model 1**	**Model 2**
**Boys**		
Ethnic Dane	1	1
Descendant	2.08 (1.46-3.05)	1.97 (1.33-2.94)
Immigrant	1.54 (1.00-2.39)	1.54 (0.99-2.44)
*Type 3 analysis*	*0.0001*	*0.0010*
**Stomach-ache**		4.91 (3.95-6.10)
**Girls**		
Ethnic Dane	1	1
Descendant	1.38 (1.03-1.88)	1.24 (0.91-1.74)
Immigrant	1.55 (1.14-2.11)	1.55 (1.12-2.15)
*Type 3 analysis*	*0.0035*	*0.0155*
**Stomach-ache**		5.18 (4.37-6.14)

Model 1: adjusted for age group (ICC = 0.0093 for boys; 0.0061for girls).

Model 2: adjusted for age group, and stomach-ache (ICC = 0.0082 for boys; 0.0060 for girls).

## Discussion

This study examined the relationship between immigrant background and medicine use for headache and stomach-ache, and the mediating effect of symptoms of headache and stomach-ache among 11-, 13- and 15-year-old adolescents living in Denmark in 2006. We found that the risk of medicine use for aches was higher for immigrants and even more so for descendant boys as compared to ethnic Danes, with the exception of medicine use for headache among girls. There were no major differences in symptoms of headache and stomach-ache between ethnic Danes and immigrants. When adjusting for symptoms the association between medicine use and immigrant background only attenuated slightly, indicating that this potential mediating factor do not explain the higher medicine use among immigrants and descendants.

### Symptoms

While aches represent the most common reason for medicine use for aches, not all medicine use for aches can be explained by prevalence of headache or stomach-ache [13]. These results are in line with what Turunen et al., who found frequent use of analgesics among participants with low pain symptom prevalence [14]. It may be that immigrant and descendant adolescents’ boys use medicines for aches outside indication to cope with psychosocial stressors in their daily lives.

### Comparison to other studies

Our findings about a higher prevalence of medicine use among immigrants and descendants correspond with a study based on an earlier HBSC data collection [15]. Two other studies show the opposite result, a study from Germany which found that self-medication was not related to immigrant background [9] and a Norwegian study which showed that there were no differences in the dispensing of prescribed analgesics between descendant adolescents and ethnic Norwegians [16]. The two latter studies, however, do not provide insight into the common over-the-counter (OTC) medicine use for aches among adolescents. Research suggests that OTC medicines may be the ones most used by young people to alleviate pain as well as discomfort and stress, especially among young women [17-20].

One of the main hypotheses in studies of migration and health is that differences by immigrant background converge over time, i.e. should be less visible among descendants than among immigrants [21]. Although our results suggest this gradient for medicine use for stomach-ache among girls, we did not find it among boys, with descendant adolescents using more medicine for aches than immigrants compared to ethnic Danes. We recommend future qualitative research to look into the reason of the highest using of medicine for aches among descendants.

### Strengths and Limitations

Our study was based on a large, national representative survey with a high response rate, which limits a potential selection bias. If the 11% non participants suffer more aches and have an over-representation of immigrants, our analyses may have under-estimated the association between immigrant background and medicine us. Although the study population was large, the number of immigrants and descendants was fairly low and precluded studies of specific immigrant populations. The focus on the crude classification into immigrants and descendants, and the choice of only medicine use for aches, resulted in sufficient statistical power for the examination of the association between immigrant background and medicine use for pain. However, as cultural background may affect medicine use behaviour, we encourage future research to examine adolescents’ use of medicine for aches among specific immigrant minority groups. Moreover, we did not include questions related to the length of stay, the citizenship or the characteristics of the healthcare system of the country of origin, and we suggest including these variables in future studies of immigrant background and medicine use.

Validation studies suggest that the measurement of symptoms and the measurement of medicine use are appropriate in age-equivalent populations [13]. However, future validation studies may need to examine the appropriateness among different immigrant groups.

## Conclusion

We found that, among adolescents in Denmark, the risk of medicine use for headache and stomach-ache was higher for immigrants and descendants as compared to ethnic Danes, with the exception of medicine use for headache among girls. When adjusting for symptoms the association attenuated only slightly, i.e. this potential mediating factor did not explain the higher medicine use among immigrants and descendants. Our results suggest that immigrant and descendant adolescents are at-risk groups regarding medicine use for aches that deserves special attention.
